# Supplementary material for: Optimized SMRT-UMI protocol produces highly accurate sequence datasets from diverse populations – application to HIV-1 quasispecies
Source: bioRxiv. 2023 Feb 24:2023.02.23.529831. Preprint. [Version 1] doi: 10.1101/2023.02.23.529831 (PMC9980183; doi:10.1101/2023.02.23.529831)

bioRxiv preprint doi: https://doi.org/10.1101/2023.02.23.529831; this version posted February 24, 2023. The copyright holder for this preprint (which was not certified by peer review) is the author/funder. This article is a US Government work. It is not subject to copyright under 17 USC 105 and is also made available for use under a CC0 license.

Supporting information

Optimized SMRT-UMI protocol produces highly accurate sequence datasets from diverse populations – application to HIV-1 quasispecies

Optimized protocol for accurately sequencing diverse populations

Dylan H. Westfall[1]
Wenjie Deng[1]
Alec Pankow[1^]
Hugh Murrell[2]
Lennie Chen[1]
Hong Zhao[1]
Carolyn Williamson[2]
Morgane Rolland[3,4]
Ben Murrell[5]
James I. Mullins[1,6,7¶]

Departments of [1]Microbiology, [6]Medicine, and [7]Global Health, University of Washington Schools of Medicine and Public Health, Seattle, WA 98195-8070 US
[2]Division of Medical Virology, Department of Pathology, University of Cape Town and National Health Laboratory Services, Cape Town, South Africa.
[3]US Military HIV Research Program, Walter Reed Army Institute of Research, Silver Spring, Maryland, 20910, USA.
[4]The Henry M. Jackson Foundation for the Advancement of Military Medicine, Inc., Bethesda, Maryland, 20817, USA.
[5]Department of Microbiology, Tumor and Cell Biology, Karolinska Institutet, 17717 Stockholm, SE

[^]Department of Microbiology, Icahn School of Medicine at Mount Sinai, New York, NY 10029, USA
[¶]Corresponding author at jmullins@uw.edu, 1-206-897-6163

bioRxiv preprint doi: https://doi.org/10.1101/2023.02.23.529831; this version posted February 24, 2023. The copyright holder for this preprint (which was not certified by peer review) is the author/funder. This article is a US Government work. It is not subject to copyright under 17 USC 105 and is also made available for use under a CC0 license.

S1 Table. Biological samples and PCR conditions. (A) Four participants with varying RNA copies numbers per ml of plasma (viral loads) had the number of PCR-amplifiable copies of 3kb amplicons estimated per PCR using QUALITY{Rodrigo, 1997 #11306} (https://indra.mullins.microbiol.washington.edu/quality/). (B) Thirty-three samples were generated from these samples using different purification method of the 1st round PCR, and total cycle numbers. Also listed are the actual template numbers recovered after sequencing (sUMI families) and any PCR primers specific to a particular participant (primer sequences provided in Supplementary Table S2).

A.

Participant	RNA c/ml	cDNA Primer	Sample ID
A	3,162,278	PB_A5_R9013	5
B	1,737,801	PB_A14_R9013	14
C	371,535	PB_A9_R9013	9
D	1,023,293	PB13_R9165	13

B.

**Sample**	**Estimated Templates in PCR**	**1st Rd PCR Rxns**	**Purification Method**	**Total PCR Cycles**	**Actual Templates in PCR**	**Forward Index**	**Reverse Index**
**Participant A**							
1	25	10	None	47	21	F01	R01
2	25	10	Ampure XP Bead	47	21	F02	R02
3	25	10	Blue Pippin	47	21	F03	R03
4	50	5	None	47	42	F04	R04
5	50	5	Ampure XP Bead	47	42	F05	R01
6	50	5	Blue Pippin	47	42	F06	R02
7	100	2	None	47	98	F07	R03
8	100	2	Ampure XP Bead	47	98	F08	R04
9	100	2	Blue Pippin	47	98	F09	R01
10	100	2	Blue Pippin	45	98	F10	R02
**Participant B**							
11	25	4	None	47	65	F01	R01
12	25	4	Ampure XP Bead	47	65	F02	R02
13	25	4	Blue Pippin	47	65	F03	R03
14	50	2	None	47	126	F04	R04
15	50	2	Ampure XP Bead	47	126	F05	R01
16	50	2	Blue Pippin	47	126	F06	R02
17	100	1	None	47	278	F07	R03
18	100	1	Ampure XP Bead	47	278	F08	R04
19	100	1	Blue Pippin	47	278	F09	R01
20	100	1	Blue Pippin	45	278	F10	R02
**Participant C**							
21	25	4	None	47	35	F01	R01
22	25	4	Ampure XP Bead	47	35	F02	R02
23	25	4	Blue Pippin	47	35	F03	R03
24	50	2	None	47	73	F04	R04
25	50	2	Ampure XP Bead	47	73	F05	R01
26	50	2	Blue Pippin	47	73	F06	R02
27	100	1	None	47	158	F07	R03
28	100	1	Ampure XP Bead	47	158	F08	R04
29	100	1	Blue Pippin	47	158	F09	R01
30	100	1	Blue Pippin	45	158	F10	R02
**Participant D**							
28	25	5	None	47	32	F01	R01
29	25	5	Ampure XP Bead	47	32	F02	R02
30	25	5	Blue Pippin	47	32	F03	R03

bioRxiv preprint doi: https://doi.org/10.1101/2023.02.23.529831; this version posted February 24, 2023. The copyright holder for this preprint (which was not certified by peer review) is the author/funder. This article is a US Government work. It is not subject to copyright under 17 USC 105 and is also made available for use under a CC0 license.

S2 Table. cDNA and PCR primer sequences. Name, usage, and sequence are listed for each primer. For each cDNA primer, one version is prepared with each of the 16 Sample ID sequences (indicated by XXXXXX).

Primer	Usage	Sequence (5'-3')
PB_AX_R9013	cDNA primer, Sample ID (red), UMI (N), HIV-specific sequence (**purple**)	**CCCGCGTGGCCTCCTGAATTAT***CCGCTCCGTCCGACGACTCACTATA*XXXXXXNNNNNNNN**GTCATTGGTCTTAAAGGTACCTG**
PBX_R9165	Alternate cDNA primer	**CCCGCGTGGCCTCCTGAATTAT***CCGCTCCGTCCGACGACTCACTATA*XXXXXXNNNNNNNN**CTGGTGTGTARTTYTGCCAATCAG**
illu_A_F5982	2nd strand primer with UMI (N) for dUMI reactions	**AATGATACGGCGACCACCGA***GATCTACACTCTTTCCCTACACG*ACNNNNNNNNN**TAGGCATCTCCTATGGCAGGAAGAAG**
PB-R1-alt1	1st round reverse primer	**CCCGCGTGGCCTCCTGAATTAT**
PB-R2-alt1	2nd round reverse primer	*CCGCTCCGTCCGACGACTCACTATA*
PB-R2-alt1_ILL	2nd round reverse primer – Index Primer binding sequence (**Blue**)	**GTCTCGTGGGCTCGGCCGCTCCGTCCGACGACTCACTATA**
illu_F1	1st rd forward primer with dUMI protocol	AATGATACGGCGACCACCGA
illu_F2	2nd rd forward primer with dUMI protocol	GATCTACACTCTTTCCCTACACG
illu_F2_ILL	2nd rd forward primer with dUMI protocol – Index Primer binding sequence (**Blue**)	TCGTCGGCAGCGTCGATCTACACTCTTTCCCTACACG
F5876	1st rd forward primer with sUMI protocol, dUMI forward primer for positive control	TAGAGCCCTGGAAGCATCCAGGAAG
F5982A	2nd rd forward primer with sUMI protocol, dUMI forward primer for positive control	TAGGCATCTCCTATGGCAGGAAGAAG
R9165	1st rd reverse primer, spiked into positive control	CTGGTGTGTARTTYTGCCAATCAG
R9013	2nd rd reverse primer, spiked into positive control	GTCATTGGTCTTAAAGGTACCTG

Index Primer	Usage	Sequence (5'-3')
F01	Forward Index Primer with Illumina Nextera ID N701	CTACACTCGCCTTATCGTCGGCAGCGTC
F02	Forward Index Primer with Illumina Nextera ID N702	CTACACCTAGTACGTCGTCGGCAGCGTC
F03	Forward Index Primer with Illumina Nextera ID N703	CTACACTTCTGCCTTCGTCGGCAGCGTC
F04	Forward Index Primer with Illumina Nextera ID N704	CTACACGCTCAGGATCGTCGGCAGCGTC
F05	Forward Index Primer with Illumina Nextera ID N705	CTACACAGGAGTCCTCGTCGGCAGCGTC
F06	Forward Index Primer with Illumina Nextera ID N706	CTACACCATGCCTATCGTCGGCAGCGTC
F07	Forward Index Primer with Illumina Nextera ID N707	CTACACGTAGAGAGTCGTCGGCAGCGTC
F08	Forward Index Primer with Illumina Nextera ID N710	CTACACCAGCCTCGTCGTCGGCAGCGTC
F09	Forward Index Primer with Illumina Nextera ID N711	CTACACTGCCTCTTTCGTCGGCAGCGTC
F10	Forward Index Primer with Illumina Nextera ID N712	CTACACTCCTCTACTCGTCGGCAGCGTC
F11	Forward Index Primer with Illumina Nextera ID N714	CTACACTCATGAGCTCGTCGGCAGCGTC
F12	Forward Index Primer with Illumina Nextera ID N715	CTACACCCTGAGATTCGTCGGCAGCGTC
F13	Forward Index Primer with Illumina Nextera ID N716	CTACACTAGCGAGTTCGTCGGCAGCGTC
F14	Forward Index Primer with Illumina Nextera ID N718	CTACACGTAGCTCCTCGTCGGCAGCGTC
F15	Forward Index Primer with Illumina Nextera ID N719	CTACACTACTACGCTCGTCGGCAGCGTC

bioRxiv preprint doi: https://doi.org/10.1101/2023.02.23.529831; this version posted February 24, 2023. The copyright holder for this preprint (which was not certified by peer review) is the author/funder. This article is a US Government work. It is not subject to copyright under 17 USC 105 and is also made available for use under a CC0 license.

F16	Forward Index Primer with Illumina Nextera ID N720	CTACACAGGCTCCGTCGTCGGCAGCGTC
F17	Forward Index Primer with Illumina Nextera ID N721	CTACACGCAGCGTATCGTCGGCAGCGTC
F18	Forward Index Primer with Illumina Nextera ID N722	CTACACCTGCGCATTCGTCGGCAGCGTC
F19	Forward Index Primer with Illumina Nextera ID N723	CTACACGAGCGCTATCGTCGGCAGCGTC
F20	Forward Index Primer with Illumina Nextera ID N724	CTACACCGCTCAGTTCGTCGGCAGCGTC
F21	Forward Index Primer with Illumina Nextera ID N726	CTACACGTCTTAGGTCGTCGGCAGCGTC
F22	Forward Index Primer with Illumina Nextera ID N727	CTACACACTGATCGTCGTCGGCAGCGTC
F23	Forward Index Primer with Illumina Nextera ID N728	CTACACTAGCTGCATCGTCGGCAGCGTC
F24	Forward Index Primer with Illumina Nextera ID N729	CTACACGACGTCGATCGTCGGCAGCGTC
R01	Reverse Index Primer with Illumina Nextera ID S502	CGAGATCTCTCTATGTCTCGTGGGCTCGG
R02	Reverse Index Primer with Illumina Nextera ID S503	CGAGATTATCCTCTGTCTCGTGGGCTCGG
R03	Reverse Index Primer with Illumina Nextera ID S505	CGAGATGTAAGGAGGTCTCGTGGGCTCGG
R04	Reverse Index Primer with Illumina Nextera ID S506	CGAGATACTGCATAGTCTCGTGGGCTCGG

**Sample ID**	**Usage**	**Sequence (5'-3')**
1	Sample ID within sUMI primer	ACAGTG
2	Sample ID within sUMI primer	CACTCA
3	Sample ID within sUMI primer	GGTAGC
4	Sample ID within sUMI primer	TAGCTT
5	Sample ID within sUMI primer	CTATAC
6	Sample ID within sUMI primer	ATCACG
7	Sample ID within sUMI primer	ACTGAT
8	Sample ID within sUMI primer	TGACCA
9	Sample ID within sUMI primer	GCTCAT
11	Sample ID within sUMI primer	CGATGT
12	Sample ID within sUMI primer	ATGCTG
13	Sample ID within sUMI primer	ACGATC
14	Sample ID within sUMI primer	GTCATC
15	Sample ID within sUMI primer	CGAGTA
16	Sample ID within sUMI primer	GACAGA
17	Sample ID within sUMI primer	TAGAGC

bioRxiv preprint doi: https://doi.org/10.1101/2023.02.23.529831; this version posted February 24, 2023. The copyright holder for this preprint (which was not certified by peer review) is the author/funder. This article is a US Government work. It is not subject to copyright under 17 USC 105 and is also made available for use under a CC0 license.

S1 Figure. Gel electrophoresis and analysis of products according to purification method and PCR cycle number. 1% agarose gel image of purified PCR products after the 5 cycle Index PCR for different initial template inputs, methods of 1st round PCR purification, and total cycle numbers.

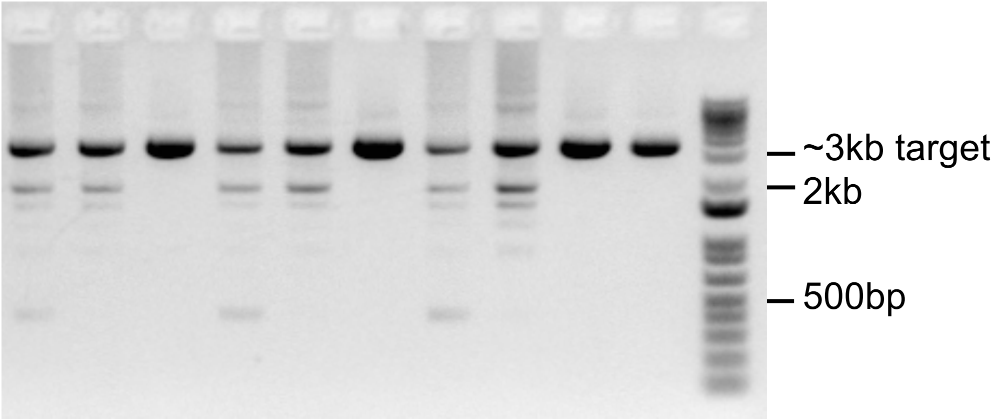

Key:

Lane	Estimated Input Templates per PCR	Purification of 1st round PCR	Total Cycles
1	25	None	47
2	25	Ampure XP Bead	47
3	25	Blue Pippin	47
4	50	None	47
5	50	Ampure XP Bead	47
6	50	Blue Pippin	47
7	100	None	47
8	100	Ampure XP Bead	47
9	100	Blue Pippin	47
10	100	Blue Pippin	45

bioRxiv preprint doi: https://doi.org/10.1101/2023.02.23.529831; this version posted February 24, 2023. The copyright holder for this preprint (which was not certified by peer review) is the author/funder. This article is a US Government work. It is not subject to copyright under 17 USC 105 and is also made available for use under a CC0 license.

S2 Figure. Analysis Flowchart. Beginning with the CCS read file from PacBio sequencing, the data moves through a series of pipelines, custom code, analysis steps, and software to create all tables and figures.

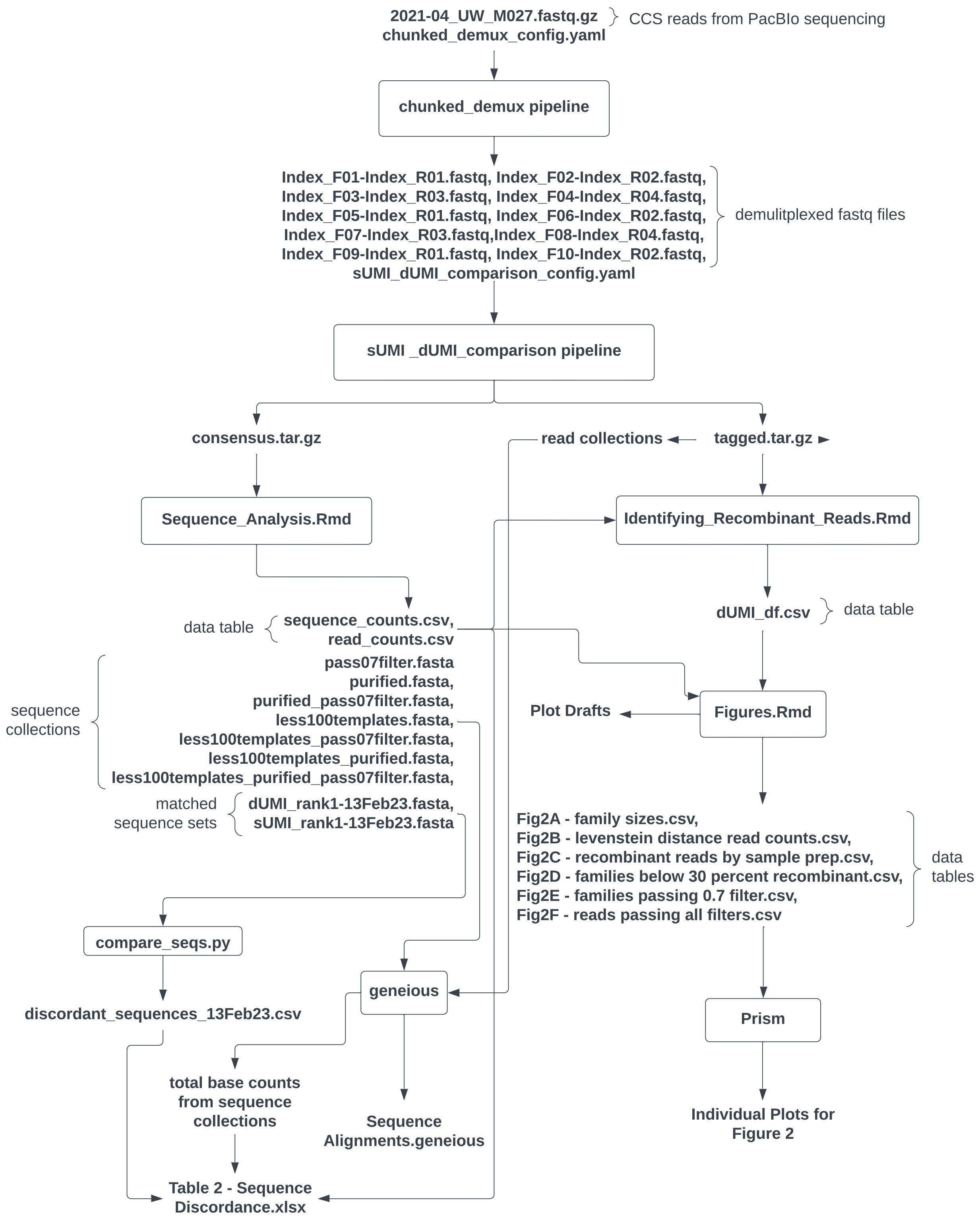

Supplement: 1 — S1 Table. Biological Samples. Thirty-three samples were generated from 4 four participants with varying viral loads by varying estimated input templates per PCR using QUALITY[24] (https://indra.mullins.microbiol.washington.edu/quality/), purification method of the 1st round PCR, and total cycle numbers. Also listed are the actual template numbers recovered after sequencing (sUMI families) and any PCR primers specific to a particular participant (primer sequences provided in S2 Table). S2 Table. cDNA and PCR primer sequences. Name, usage, and sequence are listed for each primer. For each cDNA primer, one version is prepared with each of the 16 Sample ID sequences (indicated by XXXXXX). S1 Figure. Gel electrophoresis and analysis of products according to purification method and PCR cycle number. 1% agarose gel image of purified PCR products after the 5 cycle Index PCR for different initial template inputs, methods of 1st round PCR purification, and total cycle numbers. S2 Figure. Analysis Flowchart. Beginning with the CCS read file from PacBio sequencing, the data moves through a series of pipelines, custom code, analysis steps, and software to create all tables and figures. [file NIHPP2023.02.23.529831V1-supplement-1.pdf]
